# 3D Printing of Dapagliflozin Containing Self-Nanoemulsifying Tablets: Formulation Design and In Vitro Characterization

**DOI:** 10.3390/pharmaceutics13070993

**Published:** 2021-06-30

**Authors:** Mohammed S. Algahtani, Abdul Aleem Mohammed, Javed Ahmad, M. M. Abdullah, Ehab Saleh

**Affiliations:** 1 Department of Pharmaceutics, College of Pharmacy, Najran University, Najran 11001, Saudi Arabia; msalqahtane@nu.edu.sa (M.S.A.); aaleem@nu.edu.sa (A.A.M.); 2 Promising Centre for Sensors and Electronic Devices (PCSED), Department of Physics, College of Arts and Science, Najran University, Najran 11001, Saudi Arabia; mmalsyed@nu.edu.sa; 3 Future Manufacturing Processes Research Group, Faculty of Engineering and Physical Sciences, University of Leeds, Leeds LS2 9JT, UK; E.Saleh@leeds.ac.uk

**Keywords:** dapagliflozin propanediol monohydrate, semisolid extrusion-based 3D printing, self-nanoemulsifying tablet, nanoemulsion, SEM-EDS analysis, drug dissolution

## Abstract

The 3D printing techniques have been explored extensively in recent years for pharmaceutical manufacturing and drug delivery applications. The current investigation aims to explore 3D printing for the design and development of a nanomedicine-based oral solid dosage form of a poorly water-soluble drug. A self-nanoemulsifying tablet formulation of dapagliflozin propanediol monohydrate was developed utilizing the semisolid pressure-assisted microsyringe (PAM) extrusion-based 3D printing technique. The developed formulation system consists of two major components (liquid and solid phase), which include oils (caproyl 90, octanoic acid) and co-surfactant (PEG 400) as liquid phase while surfactant (poloxamer 188) and solid matrix (PEG 6000) as solid-phase excipients that ultimately self-nanoemulsify as a drug encapsulated nanoemulsion system on contact with aqueous phase/gastrointestinal fluid. The droplet size distribution of the generated nanoemulsion from a self-nanoemulsifying 3D printed tablet was observed to be 104.7 ± 3.36 nm with polydispersity index 0.063 ± 0.024. The FT-IR analysis of the printed tablet revealed that no drug-excipients interactions were observed. The DSC and X-RD analysis of the printed tablet revealed that the loaded drug is molecularly dispersed in the crystal lattice of the tablet solid matrix and remains solubilized in the liquid phase of the printed tablet. SEM image of the drug-loaded self-nanoemulsifying tablets revealed that dapagliflozin propanediol monohydrate was completely encapsulated in the solid matrix of the printed tablet, which was further confirmed by SEM-EDS analysis. The in vitro dissolution profile of dapagliflozin-loaded self-nanoemulsifying tablet revealed an immediate-release drug profile for all three sizes (8 mm, 10 mm, and 12 mm) tablets, exhibiting >75.0% drug release within 20 min. Thus, this study has emphasized the capability of the PAM-based 3D printing technique to print a self-nanoemulsifying tablet dosage form with an immediate-release drug profile for poorly water-soluble drug.

## 1. Introduction

In the field of pharmaceutical product development, growing interest is seen in improving the solubility and dissolution profile of newly discovered drug molecules having poor aqueous solubility. The concept of developing a lipid-based formulation system for poorly water-soluble drugs has gained considerable attention as it facilitates drug solubilization and promotes the gastrointestinal absorption of encapsulated drugs [1,2].

Among various lipid-based formulation approaches, which include the formulation of liposomes [3], microemulsions [4], and nanostructured lipid carriers [5], self-nanoemulsifying drug delivery systems (SNEDDS) have proven to be the most promising approach that consists of drug molecularly dispersed in the isotropic mixture of oil, surfactant, and co-surfactant [6]. It forms a stable nanoemulsion system in contact with the aqueous phase upon oral administration. The SNEDDS upon dispersion endures self-emulsification in gastric media and encapsulates the poorly soluble therapeutics inside nano oil droplets and thereby improves the dissolution and absorption rate of the encapsulated drug (schematic illustration in Supplementary Figure S1) [7]. So far, the successfully developed and marketed SNEDDS formulations for poorly water-soluble drugs include cyclosporin A (Neoral^®^), Saquinavir (Fortovase^®^), ritonavir (Norvir^®^), etc. However, most of the currently available marketed products of SNEDDS were developed as liquid formulations, which were filled in soft gel capsules for ease of oral administration. Despite the benefits, the liquid formulations are associated with various limitations, including dose uniformity, low stability, and interaction of the liquid formulation with the capsule shell may lead to softening/brittleness of shell or change in taste perception or leakage of liquid from the filled capsule [8]. These limitations have restricted the industrial product development of liquid SNEDDS and have given a combusting interest to transform these liquid SNEDDS formulations into a solid self-nanoemulsifying dosage form. The various attempts explored to develop solid SNEDDS formulations include adsorption of liquids on solid carriers [9], spray drying and spray cooling [10,11], and extrusion/spheronization for pellet design [12]. However, these approaches are also associated with product development issues such as dose dilution [13], hindered flowability of powder formulation [14], incomplete drug release due to adsorption of liquid onto silicates [13], and tolerability/toxicity issues [15]. In recent years, 3D printing has been widely explored to overcome various pharmaceutical manufacturing issues and drug delivery challenges. The current research explores the use of 3D printing for nanomedicine-based oral formulation development of poorly soluble drugs.

3D printing is an additive manufacturing technique that enables layer-by-layer fabrication of 3D structures in a customized dose and geometry driven digitally. The interest in the field of 3D printing technology of pharmaceutical components has taken a big leap with the approval of the first 3D printed tablet of levetiracetam (SPRITAM^®^) by US-FDA in 2015 [16]. Recently in February 2021, the US FDA has given Investigational New Drug (IND) approval to Triastek for its first 3D printed drug product (T19), which is used to treat rheumatoid arthritis developed by using the 3D printing-based Melt Extrusion Deposition (MED) technique [17]. This IND approval by US-FDA is a further breakthrough towards the success and advancement of 3D printing techniques in recent years for the development of pharmaceutical products. In addition, 3D printing has been explored very recently for the formulation of SNEDDS-based lidocaine suppositories for personalized drug delivery utilizing pressure-assisted microsyringe (PAM)/extrusion-based 3D printing technique [18]. The printed suppositories composed of Geloil™ SC, Gelucire^®^ 48/16, and Kolliphor^®^ RH40 in varying composition exhibited delayed release of lidocaine and acted as a patient-tailored formulation system. In another investigation, Vithani et al. developed a solid lipid-based self-microemusifying drug delivery system for poorly soluble drugs (such as fenofibrate and cinnarizine) [19]. This study demonstrated a proof-of-concept that a 3D printing technique can be utilized to develop solid self-emulsifying drug delivery systems without using solid adsorbents to convert the liquid self-emulsifying formulation system into patient-tailored oral solid dosage forms. Furthermore, a lipid-based tablet formulation system was fabricated from emulsion gels employing the PAM-based 3D printing technique [20]. The study demonstrated the fabrication of an oil-in-water (O/W) emulsion gel for a poorly water-soluble drug (Fenofibrate) incorporated with methylcellulose, croscarmellose sodium as printable ink for the 3D printing process. The designed 3D printed tablets exhibited a rapid in vitro dispersion and digestion profile. Thus this concept provides a piece of evidence to transform a lipid-based formulation system into a solid dosage form utilizing 3D printing technique.

This work expands the use of semisolid extrusion-based 3D printing technique to develop self-nanoemulsifying tablets of a poorly water-soluble drug where Dapagliflozin propanediol monohydrate (DAP) was selected as a model drug. It is a novel sodium-glucose co-transporter-2 inhibitor (as an oral hypoglycemic agent), which is used in the management of type II diabetes and acts by preventing the reabsorption of glucose in kidneys [21]. The poor aqueous solubility and low permeability profile of DAP rationalize its formulation design as solid-SNEDDS to improve the solubilization attributes and dissolution profile exploring 3D printing technique for self-nanoemulsifying tablet design.

## 2. Materials and Method

### 2.1. Materials

Dapagliflozin propanediol monohydrate was provided by Jamjoom Pharmaceuticals, Jeddah, Saudi Arabia. Capryol 90, Poloxamer 188, Polyethylene glycol (PEG) 6000, PEG 4000, Cremophore EL, and PEG 400 were purchased from Sigma Aldrich (Gillingham, UK). Methanol was purchased from UFC Biotech (Riyadh, Saudi Arabia).

### 2.2. Choice of Material

Different formulation components (liquid and solid phase) were selected to design a solid self-nanoemulsifying drug delivery system exploiting 3D printing technology. The developed system consists of liquid phase containing oils and co-surfactant and solid phase containing surfactant and solid matrix. The liquid phase was chosen based on the drug solubility of DAP while the solid phase should have the ability to self-emulsify the liquid phase into nanoemulsion as well as accommodate this liquid phase in their solid matrix.

### 2.3. Preparation and Optimization of Semisolid Extrudable Paste for 3D Printing of Solid-SNEDDS

A semisolid paste of the selected liquid and solid phase for 3D printing was prepared by the fusion method. The excipients consist of solid-phase were taken in a beaker and melted at a temperature near 50 °C. After that, liquid phase excipients containing the drug were added to this melted phase and mixed continuously with a magnetic stirrer to form a clear homogeneous mass in a form of extrudable paste for the extrusion-based 3D printing (pressure-assisted microsyringe−PAM) technique. This homogenous semi-solid paste is then transferred to the extruder syringe and left to solidify at room temperature for 30 min. To initialize the printing process, the temperature of the extruder syringe was set to 45 °C for 10 min to attain thermal equilibrium wherein the solid mass inside the extruder syringe gets ready as an extrudable paste for 3D printing. The consistency of the paste was optimized for extrudability behavior and different process parameters (like nozzle size, printing speed, printing pressure, and printing temperature) for 3D printing of solid-SNEDDS.

### 2.4. 3D Printing of Solid-SNEDDS as a Tablet

The geometry of the 3D printed self-nanoemulsifying tablet was designed by Autodesk CAD (Computer-Aided Design) software. The design was exported as an STL (Standard Triangle Language) file and sliced through repetier software to form a printer readable G-code. The solid-SNEDDS was designed in a form of self-nanoemulsifying circular tablets of different diameters (8 mm, 10 mm, and 12 mm) utilizing a 3D printer (Biobot 1) based on the PAM technique.

### 2.5. Characterization of the 3D Printed Self-Nanoemulsifying Tablet

The 3D printed self-nanoemulsifying tablet was characterized for the following parameters:

#### 2.5.1. Determination of Size and Weight

The size of the 3D printed self-nanoemulsifying tablets was measured using a digital vernier caliper. The weight of individual 3D printed self-nanoemulsifying tablets was measured to determine any weight variability. The weight variation of the 3D printed self-nanoemulsifying tablets was determined according to the European Pharmacopeia (2.9.5) monograph [22].

#### 2.5.2. Solid State Characterization

##### Attenuated Total Reflection-Fourier Transforms Infrared Spectroscopy (ATR-FTIR)

ATR-FTIR was performed to detect the drug-excipients interaction during the formulation of self-nanoemulsifying tablets exploiting the 3D printing technique. The ATR-FTIR spectra were obtained for the pure drug, placebo, and drug-loaded self-nanoemulsifying tablets using an ATR-FTIR spectrometer (Agilent Cary 630 FTIR, Agilent Technologies, Danbury, CT, USA). A small amount (10 mg) of the sample was taken and analyzed between 400–4000 cm^−1^ to assess the chances of any interactions between drug and formulation excipients [23].

##### Powder X-ray Diffractometry (XRD)

The crystalline behavior of pure drug and physical state of DAP inside developed self-nanoemulsifying tablets were determined by powder X-ray diffractometer (PW 3040/60, PANalytical, Almelo, Netherlands) and patterns were recorded at 2*θ* in range of 3–100° with scanning speed 0.5°/min at room temperature [23].

##### Differential Scanning Calorimetry (DSC)

Thermal behavior of pure drug, placebo, and drug-loaded self-nanoemulsifying tablets were performed using Differential Scanning Calorimeter (TA DSC 25, TA Instruments, New Castle, DE, USA). An accurately weighed amount of sample (5 mg) was placed in a Tzero aluminum pan and sealed in a nitrogen environment. The nitrogen flow rate was maintained at 50 mL/min with a heating rate of 10 °C/min utilizing an empty Tzero aluminum pan as a reference [23].

#### 2.5.3. Surface Morphology by Scanning Electron Microscopy (SEM)

The solid-state surface characterization of pure drug, placebo, and drug-loaded self-nanoemulsifying tablets were determined by high-resolution field emission scanning electron microscopy (JSM-7600F, JEOL, Tokyo, Japan) operated at an accelerating voltage range of 2.0 to 5.0 kV. The sample powder was placed with the help of double-sided adhesive glue provided with conductive gold coating. The elemental compositions of the developed formulation as a drug-loaded delivery system were further confirmed by the energy-dispersive X-ray spectroscopy (EDS) [24].

#### 2.5.4. Determination of Droplet Size, Polydispersity Index (PdI), and Zeta Potential (*ξ*)

The 3D printed drug-loaded self-nanoemulsifying tablet was dispersed in distilled water and characterized to determine the droplet size, polydispersity index (PdI), and zeta potential (*ξ*) by dynamic light scattering technique using Zetasizer (Malvern Instruments Ltd., Malvern, UK) at room temperature. The measurements were carried out in triplicate. [25]

#### 2.5.5. Determination of Percentage of Drug Content

The percentage of drug content (% drug) of 3D printed self-nanoemulsifying tablets of each size was determined by dissolving the 3D printed tablets in methanol followed by further dilutions to analyze through a UV spectrophotometer at *λ*_max_ of 224 nm. The percentage of drug content of each tablet size was determined. The drug content uniformity was assessed according to the European Pharmacopeia (2.9.6) monograph to know the uniformity of content in the customized dose of tablet printed through PAM-based 3D printing technique [26].

### 2.6. In Vitro Dissolution Study

The in vitro dissolution study was performed for 3D printed drug-loaded self-nanoemulsifying tablets of each size using USP dissolution apparatus (type II). The tablets were placed in 900 mL of the simulated gastric fluid (0.1 N HCl) as dissolution media at pH 1.2. The dissolution study was performed at a temperature of 37 ± 0.5 °C and stirring speed at 50 rpm under sink conditions. The 5 mL of aliquots were withdrawn at a regular interval of 1, 3, 5, 10, 15, 20, 25, 30, 45, and 60 min. The samples were filtered and analyzed through UV spectrophotometry at *λ*_max_ of 224 nm. The drug release kinetics of 3D printed self-nanoemulsifying tablets were determined through the best-fit model [27].

## 3. Results and Discussion

### 3.1. Choice of Materials

The formulation components were selected to develop solid-SNEDDS as a self-nanoemulsifying tablet. The developed formulation was composed of liquid phase consist of oil system and co-surfactant while excipients of solid-phase serve both the purpose of solidifying and emulsifying agent.

#### 3.1.1. Selection of Liquid Phase

The liquid phase was selected based on the solubilizing capacity of DAP in different oil systems. Among the tested oil systems like oleic acid, olive oil and peanut oil exhibited less solubility of DAP (≤5 mg/mL) whereas capryol 90 and octanoic acid exhibited better solubility of DAP 15.5 ± 1.24 mg/mL and 15.8 ± 1.06 mg/mL, respectively. The spontaneous nanoemulsion formation of a SNEDDS is facilitated by the addition of a co-surfactant to the system, which helps to minimize the interfacial tension between the aqueous and oily phases [28,29]. PEG 400 has been incorporated as a co-surfactant in the liquid phase as it also exhibited desirable solubility of DAP (22.45 ± 1.12 mg/mL) to further improve the drug loading. A combination of capryol 90, octanoic acid, and PEG 400 has been selected in a ratio of 2:2:1 as a liquid phase system for the development of solid-SNEDDS as a self-nanoemulsifying tablet exploiting 3D printing.

#### 3.1.2. Selection of Solid Phase

The solid phase was selected based on its ability to self-nanoemulsify the chosen liquid phase system along with the capability to accommodate the liquid phase system in their crystal lattice. The mixture of poloxamer 188 along with PEG 6000 in a ratio of 1:1 was selected as the choice of surfactant and solidifying matrix for the chosen liquid phase. It exhibited self-dispersibility and has the ability to self-nanoemulsify the chosen liquid system into nanoemulsion in contact with the aqueous phase. Poloxamer 188, due to its surface-active properties, has also been employed as a solubility enhancing solid dispersion carrier for poorly water-soluble drugs [30]. The hydrophilicity and hydrophile-lipophile balance (HLB) value of poloxamer 188 (HLB value = 29) make it a suitable candidate to form an O/W nanoemulsion system [31]. Also, the addition of PEG 6000 along with poloxamer 188 as a solid matrix system for chosen liquid phase is helpful to reduce the overall absolute amount of poloxamer 188 being a solidifying agent [32]. Therefore, a combination of poloxamer 188 along with PEG 6000 in the ratio of 1:1 has been optimized as a solid phase system to develop solid-SNEDDS as a self-nanoemulsifying tablet exploiting 3D printing technology.

### 3.2. Preparation and Optimization of Semisolid Extrudable Paste for 3D Printing of Solid-SNEDDS

#### 3.2.1. Preparation of the Paste

A semisolid extrudable paste of the optimized composition of liquid and solid phase in the ratio of 1:1.5 was prepared by fusion method with continuous stirring near to 50 °C. The percentage composition of solid-SNEDDS as the self-nanoemulsifying tablet is shown in Table 1.

**Figure 1:**
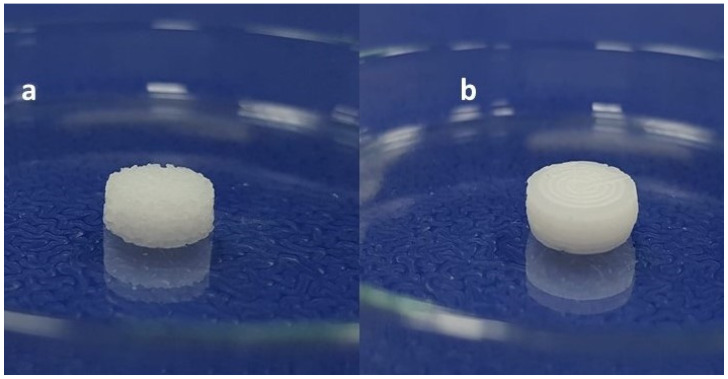
Illustration depicting the effect of mixing on extrudable paste optimized for the 3D printing of self-nanoemulsifying tablet through the PAM-based technique (**a**) Self-nanoemulsifying tablet (appearance of the surface is rough and granular) printed through the mixing of liquid and solid phase by fusion method without continuous stirring. (**b**) Self-nanoemulsifying tablet (appearance of the surface is smooth and uniform) printed through the mixing of liquid and solid phase by fusion method with continuous stirring.

The influence of mixing of liquid and solid phase of SNEDDS composition through fusion method with and without continuous stirring was observed and illustrated in Figure 1.

It was observed that the paste prepared without continuous mixing through magnetic stirrer caused the appearance of the rough, granular surface of the self-nanoemulsifying tablet upon 3D printing through PAM-based technique (Figure 1a). The appearance of the uneven surface of the 3D printed self-nanoemulsifying tablet occurred upon loading of the melted solution of the solid and liquid phase excipients without mixing in the extruder syringe and allowing it to solidify in the extruder syringe before 3D printing of the tablets.

To overcome this issue, continuous mixing of the melted solution of the solid and liquid phase excipients through a magnetic stirrer was carried out before loading in the extruder syringe. The melted mass of the excipients of the solid phase was uniformly mixed with the excipients of the liquid phase and the resultant liquid solution getting thicker as semisolid paste under the influence of continuous magnetic stirring at room temperature for 15 min. This paste of semisolid consistency was transferred into the extruder syringe and subjected to 3D printing under similar conditions of room temperature and process parameters. The continuous mixing of the formulation paste before filling in the extruder syringe enabled the fabrication of smooth-surfaced 3D printed tablets (Figure 1b). This investigation validates the significance of mixing for the formation of extrudable paste of semisolid consistency on the surface appearance of 3D printed self-nanoemulsifying tablets.

#### 3.2.2. Optimization of Printing Process Parameters and Design of Printed Geometry

The prepared semisolid paste was extruded through the 3D printer to optimize the process parameters and extrudability behavior. The semisolid paste was loaded into a heated extruder syringe to initiate the extrusion process for 3D printing. The printing was carried out at a temperature range starting from 30 to 50 °C. 3D printing at low temperatures starting from 30 to 40 °C resulted in hindered extrusion. This temperature range was observed to be not sufficient to extrude the loaded paste in the extruder syringe under applied pressure conditions due to a lack of consistency. At high temperatures (above 45–50 °C), the extrusion of paste was dripping with rapid flow due to liquefaction of the loaded paste with the rise of temperature. It was observed that extrusion of loaded paste showed better extrudability behavior under the influence of applied printing pressure at a temperature range between 40 to 45 °C.

The printing was carried out at varying printing pressures starting from 40–80 PSI upon thermal equilibrium (40 to 45 °C) of loaded paste for 10 min to optimize the printing pressure, which influences the material extrusion rate. Printing at low pressure exhibited slow extrusion material while printing at high pressure resulted in rapid extrusion of the loaded material [33]. Finally, based on the extrusion behavior and uniformity in the formation of printed layers, applied pressure of 60 PSI was found to be optimal for the 3D printing of solid-SNEDDS.

The process parameters for 3D printing of solid-SNEDDS were further optimized for the desirable nozzle diameter and printing speed. The nozzle diameter was optimized depending on the consistency and extrudability behavior of the loaded paste in the extruder syringe. Small diameter nozzles exhibited hindered extrusion due to the blockage of nozzle upon solidification of extruding material in the nozzle tip, whereas larger diameter nozzles resulted in increased thickness of the printed layer ultimately lowering the resolution of the 3D printed structures [34]. Therefore, based on printed line assessment, a nozzle diameter of 0.84 mm was optimal for 3D printing of the solid-SNEDDS at the previously optimized pressure and temperature conditions. Furthermore, layer printing tests were performed to optimize the printing speed of the motion of the print-head. Different extrusion behaviors were observed upon varying the printing speed (6–16 mm/s). At low speed, it resulted in over extrusion wherein very thick lines were formed with respect to nozzle size. The printed lines were found bloated in appearance and no structural fineness could be achieved with print speeds below 10 mm/s. At high speeds above 10 mm/s under extrusion was observed, which appeared as broken lines due to the nozzle moving faster than the extruded material. The printed lines thickness was also observed to be smaller than the nozzle size. Based on the observations from the layer printing tests carried out at varying speeds, a printing speed of 10 mm/s was optimized for the 3D printing of solid-SNEDDS. Printing at an optimal speed was helpful to achieve the desired extrusion rate and the thickness of the printed line was observed to be slightly more than the nozzle size. It facilitates the adhesion of the printed layers and provides a desired geometrical feature of the printed object [35]. Finally, based on the results obtained from the layer printing test (shown in Supplementary Table S1), the printed layer/line dimensions were measured (layer thickness and height), which paves the way to design the geometry of the 3D printed self-nanoemulsifying tablets.

### 3.3. 3D Printing of Solid-SNEDDS as the Self-Nanoemulsifying Tablet

3D printing of the designed geometry of self-nanoemulsifying tablets was carried out as per the pre-optimized printing conditions. The optimized conditions of the process parameters for 3D printing of the self-nanoemulsifying tablets are shown in Table 2.

**Figure 2:**
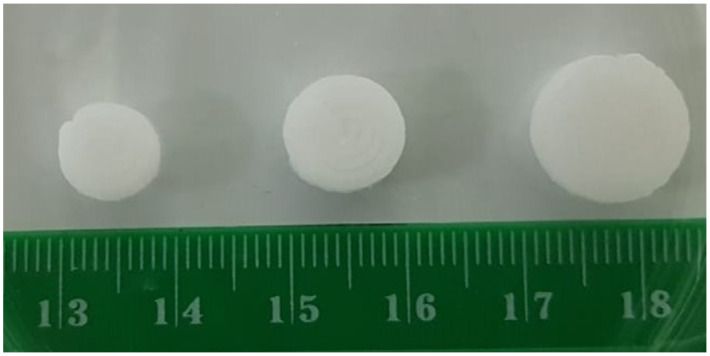
3D printed self-nanoemulsifying tablets of different sizes (8 mm, 10 mm, and 12 mm).

The self-nanoemulsifying tablets of DAP were printed in three different sizes identified as tablet A, B, and C with 8 mm, 10 mm, and 12 mm diameters, respectively. The 3D printed self-nanoemulsifying tablets of DAP of different size for dose customization is illustrated in Figure 2.

### 3.4. Characterization of the 3D Printed Self-Nanoemulsifying Tablet

#### 3.4.1. Determination of Size, Weight Variation, and % Drug Content

The 3D printed tablets were evaluated for size, weight variation, and % drug content. Table 3 shows the results of the three different sizes (A, B, and C) in terms of size (diameter and height) as 8 mm × 3 mm, 10 mm × 3 mm, and 12 mm × 3 mm, respectively. The average weights of tablets A, B, and C were found to be 193.36 ± 4.2 mg, 277.96 ± 3.6 mg, and 439.76 ± 6.2 mg respectively. The average drug contents of tablets A, B, and C were found to be 3.09 ± 0.05 mg, 5.00 ± 0.07 mg, and 7.03 ± 0.09 mg, respectively. The results of the weight variation test for all three sizes were found to be within the pharmacopeial limits (±7.5% for 12 mm size tablets and ±5% for 10 mm and 8 mm size tablets) [22,26]. It was observed that the %drug content for all three sizes 3D printed self-nanoemulsifying tablets of DAP were found to be within the pharmacopeial limit (99.46 ± 0.47, 99.12 ± 0.24, and 99.4 ± 0.09 for tablet size of 8 mm, 10 mm, and 12 mm, respectively).

#### 3.4.2. Solid State Characterization

##### Attenuated Total Reflection-Fourier Transforms Infrared Spectroscopy (ATR-FTIR)

**Figure 3:**
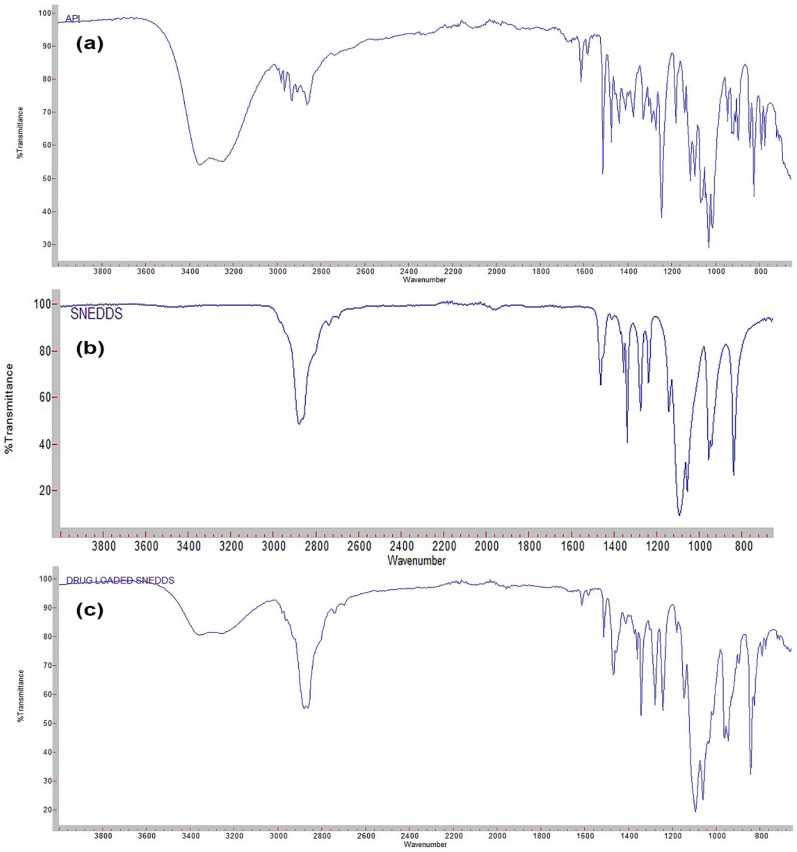
FT-IR spectra of (**a**) DAP; (**b**) placebo self-nanoemulsifying tablet; (**c**) self-nanoemulsifying tablet of DAP.

The FTIR spectra of DAP, placebo, and drug-loaded 3D printed self-nanoemulsifying tablets were analyzed to observe the possibility of any interaction between the drug-excipients (Figure 3). The FTIR spectra of DAP exhibited characteristic absorption peaks at 3350.14 cm^−1^, 2860.11cm^−1^, 1611.08 cm^−1^, and 1243.30 cm^−1^ due to hydroxyl OH bond stretching, C–H bond stretching, aromatic C=C bond stretching, and ester C–O bond stretching, respectively. Moreover, the FTIR spectra of placebo 3D self-nanoemulsifying tablet exhibited absorption peaks at 2878.04 cm^−1^ and 1279.07 cm^−1^ representing C–H stretching and C–O stretching, respectively. The FTIR spectra of the 3D printed self-nanoemulsifying tablet of DAP exhibited all the functional group peaks represented in spectra of pure drug and placebo 3D printed self-nanoemulsifying tablet with no additional peaks. This indicates that there was no interaction between the drug and excipients utilized in the preparation of the 3D printed self-nanoemulsifying tablets [23].

##### Differential Scanning Calorimetry (DSC)

**Figure 4:**
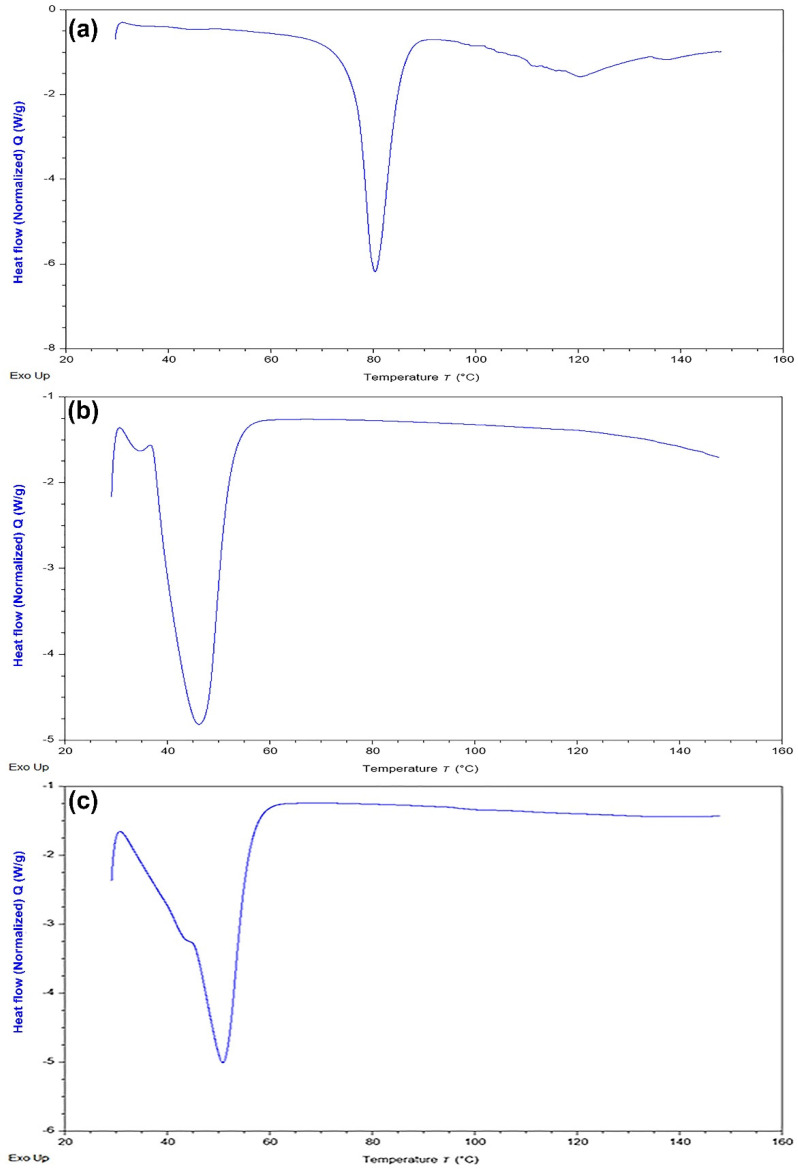
DSC thermogram of (**a**) DAP; (**b**) physical mixture of poloxamer 188 and PEG 6000; (**c**) self-nanoemulsifying tablet of DAP.

The DSC thermogram of DAP, placebo, and drug-loaded 3D printed self-nanoemulsifying tablets were analyzed and shown in Figure 4. It is observed that DAP exhibited a sharp endothermic peak at 80.6 °C, indicating the melting point of the drug and its crystalline nature. No distinct peaks were observed for the physical mixture of excipients (poloxamer 188 and PEG 6000) near this range of temperature. A sharp endothermic peak near 52 °C was observed that correlates to the melting points of the physical mixture of poloxamer 188 and PEG 6000. The DSC thermogram of the drug-loaded tablets did not show the characteristic endothermic peak of DAP. This indicates that DAP remains in the solubilized state in liquid phase components of the 3D printed self-nanoemulsifying tablets and is molecularly dispersed in the crystal lattice of the solid phase components (poloxamer 188 and PEG 6000) [36].

##### Powder X-ray Diffractometry (XRD)

**Figure 5:**
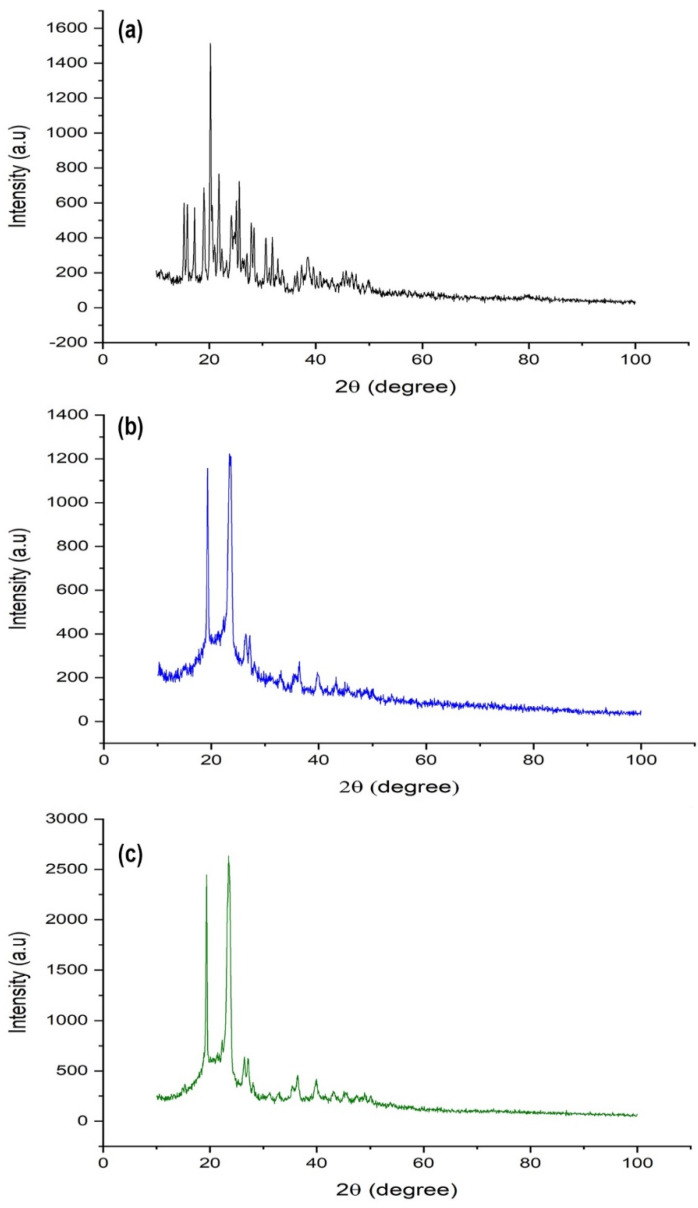
X-ray diffraction pattern of (**a**) DAP; (**b**) placebo self-nanoemulsifying tablet; (**c**) self-nanoemulsifying tablet of DAP.

The molecular dispersion state of the drug in the developed formulation system was further validated by powder-XRD analysis. The X-ray diffractograms of DAP, placebo, and drug-loaded 3D printed self-nanoemulsifying tablets were analyzed and shown in Figure 5. The typical diffraction patterns of DAP revealed a highly crystalline structure. Moreover, these typical diffraction patterns of DAP were lacking in the drug-loaded 3D printed self-nanoemulsifying tablet. This indicates that the encapsulated drug molecules remain in the solubilized state in liquid phase components of the 3D printed self-nanoemulsifying tablet and molecularly dispersed in the crystal lattice of the solid phase components (poloxamer 188 and PEG 6000) [23,36].

#### 3.4.3. Surface Morphology by Scanning Electron Microscopy (SEM)

**Figure 6:**
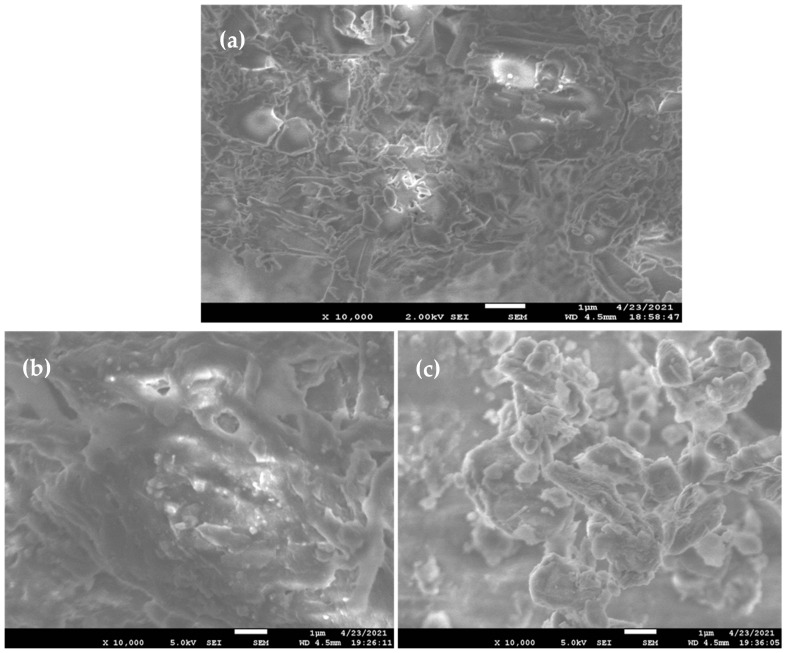
Scanning electron microscopic analysis (×10,000 magnification) (**a**) Dapagliflozin propanediol monohydrate (**b**) placebo 3D printed self-nanoemulsifying tablet (**c**) drug-loaded 3D printed self-nanoemulsifying tablet.

The surface morphology of the pure drug sample and 3D printed self-nanoemulsifying systems as placebo and drug-loaded tablets were determined through SEM analysis (Figure 6). The SEM image pure drug sample (Figure 6a) differs in appearance compared to the SEM image of the 3D printed self-nanoemulsifying tablet (Figure 6b,c). Moreover, the SEM image of the 3D printed self-nanoemulsifying tablet (Figure 6b) reveals that poloxamer 188 and PEG 6000 act as a suitable solid carrier system to accommodate the liquid phase SNEDDS into its crystal lattice through complete solidification of a mixture of liquid and solid phase components of the printed tablet. Furthermore, the SEM image of the drug-loaded self-nanoemulsifying tablet (Figure 6c) revealed that DAP is completely encapsulated in the solid matrix of poloxamer 188 and PEG 6000. The surface morphology of the DAP (Figure 6a) as inspected in SEM image analysis reveals the mosaic-like grains of varying sizes within microranges. Well-defined grain boundaries are explicitly visible in the image for almost all the grains, indicating the grain-like structure of DAP. An SEM image of the placebo (Figure 6b) and drug-loaded tablet (Figure 6c) shows the agglomerated structure of the material. Compared to the placebo tablet ((Figure 6b), the morphology of the drug-loaded tablet (Figure 6c) is almost similar and shows the agglomerated growth of the material. More accurately, the morphology of the drug-loaded tablet represents the dominating surface morphology of the placebo tablet. It indicates that the DAP has been dispersed/distributed into the lattices of placebo tablet.

**Figure 7:**
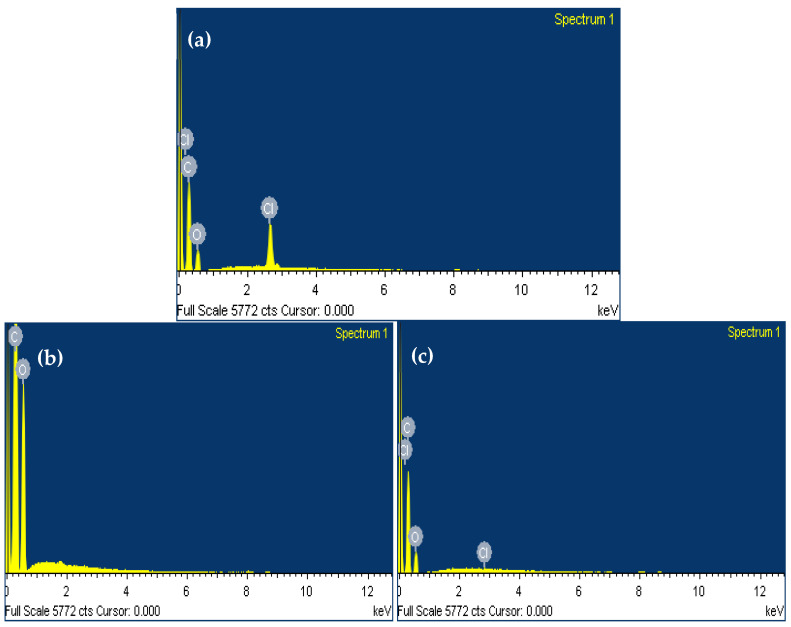
EDS analysis (**a**) spectrum of Dapagliflozin propanediol monohydrate [C_21_H_25_ClO_6_] showing the presence of carbon [C], oxygen [O] and chlorine [Cl] as characteristic peak for its elemental composition (**b**) spectrum of placebo 3D printed self-nanoemulsifying tablet showing the presence of carbon [C], and oxygen [O] as characteristic peak for its elemental composition (**c**) spectrum of drug-loaded 3D printed self-nanoemulsifying tablet showing the presence of carbon [C], oxygen [O] and chlorine [Cl] as characteristic peak for its elemental composition.

The elemental composition of 3D printed self-nanoemulsifying tablet formulation as a drug-loaded delivery system was further confirmed by EDS analysis (Figure 7c). It showed the presence of characteristics peak for chlorine in the EDS spectrum of the 3D printed self-nanoemulsifying tablet of DAP. Moreover, placebo 3D printed self-nanoemulsifying tablets lack the presence of characteristics peak for chlorine in the EDS spectrum (Figure 7b). This reveals that the developed formulation successfully accommodates the solubilized form of DAP in the crystal lattice of the printed tablet.

#### 3.4.4. Determination of Droplet Size, Polydispersibility Index (PdI), and Zeta Potential (*ξ*)

**Figure 8:**
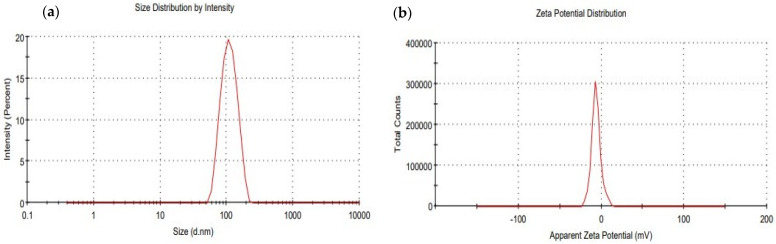
Droplet size distribution (**a**) and zeta potential (**b**) of the 3D printed self-nanoemulsifying tablet of DAP.

The droplet size distribution, PdI, and zeta potential of 3D printed self-nanoemulsifying tablets of DAP were determined by dynamic light scattering technique through zeta sizer. The average droplet size of the 3D printed self-nanoemulsifying tablets of DAP was found to be 104.7 ± 3.36 nm with a PdI value of 0.063 ± 0.024 (Figure 8a). This droplet size range of around 100 nm would improve the drug dissolution and absorption upon oral administration because smaller droplets of drug-loaded carrier systems have a greater surface area available to enhance drug absorption and systemic bioavailability [37,38]. Furthermore, the stability of the drug-loaded nanoemulsion system is subjective to the surface charge. The higher surface charge will increase electrical repulsive forces, which prevent the nanodroplets coalescence in the gastrointestinal fluid [39]. The surface charge on nanoemulsion droplets generated from 3D printed self-nanoemulsifying tablets of DAP in the presence of aqueous phase was found to have a negative polarity with zeta potential −6.84 ± 0.34 mV (Figure 8b). This indicates that the generated nanoemulsion system on self-nanoemulsification of 3D printed tablet inside the gastrointestinal tract acts as a stable drug carrier system, which would be helpful in improving the absorption profile of the encapsulated drug.

### 3.5. In Vitro Dissolution Study and Drug Release Kinetics

**Figure 9:**
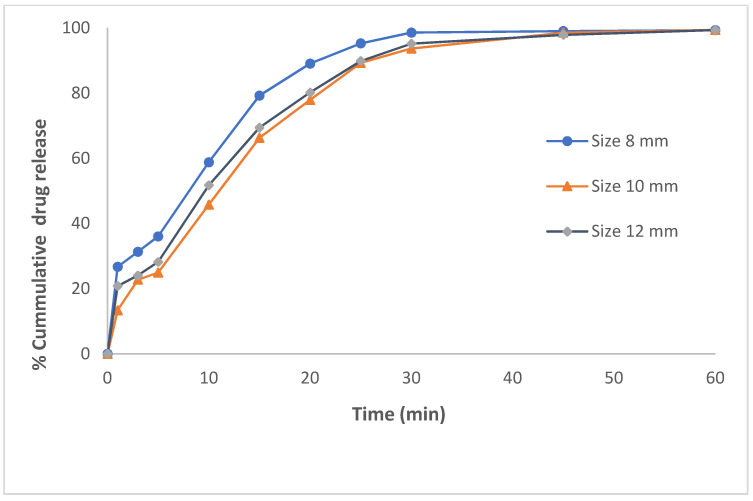
In vitro drug release profile of the 3D printed self-nanoemulsifying tablet of DAP of varying dimensions (8 mm, 10 mm, and 12 mm).

The in vitro dissolution profile of the DAP-loaded 3D printed self-nanoemulsifying tablets revealed an immediate-release drug profile for all three tablet sizes (8 mm, 10 mm, and 12 mm) as shown in Figure 9. The % cumulative drug release from 3D printed self-nanoemulsifying tablets within 25 min was 95.27 ± 0.21, 89.27 ± 0.15, and 89.87 ± 0.12 for the tablets of 8 mm, 10 mm, and 12 mm diameter, respectively. The smaller tablets (8 mm) exhibited a higher % cumulative drug release compared to larger diameter tablets (10 mm or more). This is because of the faster release rate of the smaller size tablets compared to larger ones due to the high surface area to volume ratio (SA:V) of smaller tablets. Tablets with high SA:V exhibit a higher rate of release due to the high level of interaction between the surface of the tablets and the surrounding media [40]. For 3D printed self-nanoemulsifying tablets, the SA:V of 8 mm diameter tablets is 1.16:1.00 whereas the SA:V of 10 mm and 12 mm diameter tablets is 1.06:1.00 and 1.00:1.00, respectively. Approximately 95.0% of DAP was released from all three sizes of tablets in 30 min. The use of poloxamer 188 as an emulsifying and solidifying agent did not hinder/delay the rate of self-nanoemulsification of solid-SNEDDS as the drug-lipid phase remained uniformly dispersed in the microstructure of poloxamer 188 and PEG 6000 [19], and resulted in the formation of stable nanomedicine-based solid dosage forms utilizing 3D printing technology. The traditional solidification techniques utilized solid carrier as an adsorbent (such as colloidal silica, dextran, microcrystalline cellulose, and lactose, etc.) and converted to solid SNEDDS-based pellets through spray drying, spray cooling, and extrusion-spheronization technique [41,42]. These techniques require the addition of a high amount of solid carrier to adsorb the liquid SNEDDS for their conversion into solid-SNEDDS. The addition of a higher amount of solid adsorbent hinders the self-nanoemulsification of SNEDDS and may require incorporation of a higher amount of surfactant to facilitate self-nanoemulsification and formation of colloidal dispersion upon contact with the aqueous phase [43,44]. Moreover, the addition of high amounts of adsorbent may also affect the dose uniformity, reproducibility, and raise safety concern [45,46].

The drug release kinetics model describes a particular drug release characteristics from the developed formulation system, which exhibits the actual mass transport mechanism of drug release involved to quantitatively predict the exact drug release kinetics. The drug release kinetics from 3D printed self-nanoemulsifying tablet formulation was best described by the Higuchi model. This observation aligns with the previously published literature report related to drug release kinetic behavior for a solid-SNEDDS [47]. The developed formulation system exhibited the best linearity and regression coefficient values of 0.986 with a release exponent (*n*) value of 0.191 for the tablet of 8 mm size whereas the regression coefficient and n value for the tablet of 10 mm and 12 mm diameters were 0.980 with *n* value of 0.265 and 0.985 with *n* value of 0.221, respectively. This indicates that the drug release from the developed formulation system was a square root of time. Furthermore, *n* values of release exponent of <0.5 indicate that the 3D printed self-nanoemulsifying tablet of DAP exhibited fickian diffusion. It indicates an immediate-release drug profile from a developed formulation system, without depicting any lag time [48]. Thus, the 3D printing technology was successfully utilized to design and develop nanomedicine-based solid dosage forms of DAP with immediate-release drug profiles. This investigation further extends the line of research carried out in the area of SNEDDS-based pharmaceutical product development [18,19,20] utilizing the semisolid extrusion-based 3D printing technique.

## 4. Conclusions

This work combines SNEDDS and 3D printing technology approach to enhance the biopharmaceutical attributes of DAP and provide the ability to dispense tailored doses for diabetic patients. The use of poloxamer 188, which acts as a surfactant and solidifying agent has led to the development of a solid self-nanoemulsifying tablet without using an additional adsorbent or carrier system. The developed 3D printed self-nanoemulsifying tablet in aqueous dispersion exhibited encapsulation of the drug in stable nanoemulsion system with negative surface charge. The fabricated 3D printed tablet showed no interaction of drug with formulation excipients during the printing process and the existence of the drug in a solubilized and molecularly dispersed state in the solid carrier system, which is described through solid-state characterization. The 3D printed self-nanoemulsifying tablet showed an immediate-release drug profile for all three tablet sizes (8 mm, 10 mm, and 12 mm) with improved biopharmaceutical attributes. Thus, the 3D printing technique provides an alternative to design and develop self-nanoemulsifying solid dosage forms for poorly water-soluble drugs.

## Figures and Tables

**Table 1:** Composition of 3D printed solid-SNEDDS as the self-nanoemulsifying tablet.

Formulation Ingredients	% Composition (*w/v*)
Liquid phase	
Capryol 90	16.00%
Octanoic acid	16.00%
PEG 400	8.00%
Solid phase	
Poloxamer 188	30.00%
PEG 6000	30.00%

**Table 2:** Optimized process parameters for 3D printing of self-nanoemulsifying tablets of DAP.

3D Printed SNEDDS Tablet	No. of Horizontally Printed Layers	No. of Circular Printed Layers	Printing Time	Printing Speed (mm/s)	Printing Pressure (PSI)	Nozzle Size (mm)
Tablet A	6	4	1 min 24 s	10	60	0.84
Tablet B	6	5	1 min 43 s	10	60	0.84
Tablet C	6	6	2 min 17 s	10	60	0.84

**Table 3:** Size, weight, and % drug content of 3D printed self-nanoemulsifying tablets of DAP.

3D Printed Self-Nanoemulsifying Tablets	Observed Size (Length × Height) in mm	Average Weight (mg)	% Drug Content
Tablet A (8 mm × 3 mm)	(8.108 ± 0.152) × (3.116 ± 0.103)	193.36 ± 4.2	99.46 ± 0.47
Tablet B (10 mm × 3 mm)	(10.052 ± 0.046) × (3.083 ± 0.100)	277.96 ± 3.6	99.12 ± 0.24
Tablet C (12 mm × 3 mm)	(11.983 ± 0.275) × (3.039 ± 0.075)	439.76 ± 6.2	99.4 ± 0.09

## Data Availability

The data presented in this study are available in article or Supplementary Materials.

## Supplementary Materials

The following are available online at https://www.mdpi.com/article/10.3390/pharmaceutics13070993/s1, Figure S1: Schematic illustration presenting the generation of drug enclosed nanoemulsion system upon self-nanoemulsification of 3D printed self-nanoemulsifying tablets in gastrointestinal fluid, Table S1: Optimization of extrudability behavior based on printed layer/line dimension (thickness and height).

## References

[1] Humberstone A.J., Charman W.N. (1997). Lipid-based vehicles for the oral delivery of poorly water soluble drugs. Adv. Drug Deliv. Rev..

[2] Porter C.J.H., Pouton C.W., Cuine J.F., Charman W.N. (2008). Enhancing intestinal drug solubilisation using lipid-based delivery systems. Adv. Drug Deliv. Rev..

[3] Lee M.K. (2020). Liposomes for Enhanced Bioavailability of Water-Insoluble Drugs: In Vivo Evidence and Recent Approaches. Pharmaceutics.

[4] Subongkot T., Ngawhirunpat T. (2017). Development of a novel microemulsion for oral absorption enhancement of all-trans retinoic acid. Int. J. Nanomed..

[5] Rizwanullah M., Amin S., Ahmad J. (2017). Improved pharmacokinetics and antihyperlipidemic efficacy of rosuvastatin-loaded nanostructured lipid carriers. J. Drug Target.

[6] Rehman F.U., Shah K.U., Shah S.U., Khan I.U., Khan G.M., Khan A. (2017). From nanoemulsions to self-nanoemulsions, with recent advances in self-nanoemulsifying drug delivery systems (SNEDDS). Expert Opin. Drug Deliv..

[7] Vithani K., Hawley A., Jannin V., Pouton C., Boyd B.J. (2018). Solubilisation behaviour of poorly water soluble drugs during digestion of solid SMEDDS. Eur. J. Pharm. Biopharm..

[8] Kalepu S., Manthina M., Padavala V. (2013). Oral lipid-based drug delivery systems—An overview. Acta Pharm. Sin. B.

[9] Oh D.H., Kang J.H., Kim D.W., Lee B.J., Kim J.O., Yong C.S., Choi H.G. (2011). Comparison of solid selfmicroemulsifying drug delivery system (solid SMEDDS) prepared with hydrophilic and hydrophobic solid carrier. Int. J. Pharm..

[10] Passerini N., Albertini B., Perissutti B., Rodriguez L. (2006). Evaluation of melt granulation and ultrasonic spray congealing as techniques to enhance the dissolution of praziquantel. Int. J. Pharm..

[11] Yi T., Wan J., Xu H., Yang X. (2008). A new solid self-microemulsifying formulation prepared by spray-drying to improve the oral bioavailability of poorly water soluble drugs. Eur. J. Pharm. Biopharm..

[12] Newton M., Petersson J., Podczeck F., Clarke A., Booth S. (2001). The influence of formulation variables on the properties of pellets containing a self-emulsifying mixture. J. Pharm. Sci..

[13] Nazzal S., Khan M.A. (2006). Controlled release of a self-emulsifying formulation from a tablet dosage form: Stability assessment and optimization of some processing parameters. Int. J. Pharm..

[14] Agarwal V., Siddiqui A., Ali H., Nazzal S. (2009). Dissolution and powder flow characterization of solid self-emulsified drug delivery system (SEDDS). Int. J. Pharm..

[15] Mandic J., Pobirk A.Z., Vrecer F., Gasperlin M. (2017). Overview of solidification techniques for self-emulsifying drug delivery systems from industrial perspective. Int. J. Pharm..

[16] United States Food and Drug Administration, Highlights of Prescribing Information Spritam. 2015. http://www.accessdata.fda.gov/drugsatfda_docs/label/2015/207958s000lbl.pdf.

[17] https://3dprintingindustry.com/news/triastek-receives-fda-ind-clearance-for-3d-printed-drug-to-treat-rheumatoid-arthritis-184159/.

[18] Chatzitaki A.T., Tsongas K., Tzimtzimis E., Tzetzis D., Bouropoulos N., Barmpalexis P., Eleftheriadis G., Fatouros D. (2021). 3D printing of patient-tailored SNEDDS-based suppositories of lidocaine. J. Drug Deliv. Sci. Technol..

[19] Vithani K., Goyanes A., Jannin V., Basit A.W., Gaisford S., Boyd B.J. (2019). A Proof of Concept for 3D Printing of Solid Lipid-Based Formulations of Poorly Water-Soluble Drugs to Control Formulation Dispersion Kinetics. Pharm. Res..

[20] Johannesson J., Khan J., Hubert M., Teleki A., Bergström C.A.S. (2021). 3D-printing of solid lipid tablets from emulsion gels. Int. J. Pharm..

[21] Plosker G.L. (2014). Dapagliflozin: A review of its use in patients with type 2 diabetes. Drugs..

[22] European Pharmacopoeia (2017). 2.9.5 Uniformity of Mass of Single-Dose Preparations.

[23] Singh S., Singh S.K., Vuddanda P.R., Srivastava A.K. (2013). A comparison between use of spray and freeze-drying techniques for preparation of solid self-microemulsifying formulation of valsartan and in vitro and in vivo evaluation. BioMed Res. Int..

[24] Abdullah M.M., Siddiqui S.A., Al-Abbas S.M. (2020). Physio-Chemical Properties and Dielectric Behavior of As-Grown Manganese Oxide (γ-Mn2 O3) Nanoparticles. J. Electron. Mater..

[25] Ahmad J., Kohli K., Mir S.R., Amin S. (2011). Formulation of self-nanoemulsifying drug delivery system for telmisartan with improved dissolution and oral bioavailability. J. Dispers. Sci. Technol..

[26] European Pharmacopoeia (2017). 2.9.6 Uniformity of Content of Single-Dose Preparations.

[27] De Meira R.Z.C., Maciel A.B., Murakami F.S., de Oliveira P.R., Bernardi L.S. (2017). In Vitro Dissolution Profile of Dapagliflozin: Development, Method Validation, and Analysis of Commercial Tablets. Int. J. Anal. Chem..

[28] Date A.A., Nagarsenker M.S. (2007). Design and evaluation of self-nanoemulsifying drug delivery systems (SNEDDS) for cefpodoxime proxetil. Int. J. Pharm..

[29] Kommuru T.R., Gurley B., Khan M.A., Reddy I.K. (2001). Self-emulsifying drug delivery systems (SEDDS) of coenzyme Q10: Formulation development and bioavailability assessment. Int. J. Pharm..

[30] Brüsewitz C., Schendler A., Funke A., Wagner T., Lipp R. (2007). Novel poloxamer-based nanoemulsions to enhance the intestinal absorption of active compounds. Int. J. Pharm..

[31] Rowe R.C., Sheskey P.J., Weller P.J. (2006). Handbook of Pharmaceutical Excipients.

[32] Li P., Hynes S.R., Haefele T.F., Pudipeddi M., Royce A.E., Serajuddin A.T.M. (2009). Development of clinical dosage forms for a poorly water– soluble drug II: Formulation and characterization of a novel solid microemulsion preconcentrate system for oral delivery of a poorly water–soluble drug. J. Pharm. Sci..

[33] Algahtani M.S., Mohammed A.A., Ahmad J., Saleh E. (2020). Development of a 3D printed coating shell to control the drug release of encapsulated immediate-release tablets. Polymers.

[34] Zidan A., Alayoubi A., Coburn J., Asfari S., Ghammraoui B., Cruz C.N., Ashraf M. (2019). Extrudability analysis of drug loaded pastes for 3D printing of modified release tablets. Int. J. Pharm..

[35] Mohammed A.A., Algahtani M.S., Ahmad M.Z., Ahmad J. (2021). Optimization of semisolid extrusion (pressure-assisted microsyringe)-based 3D printing process for advanced drug delivery application. Ann. 3D Print Med..

[36] Seo Y.G., Kim D.H., Ramasamy T., Kim J.H., Marasini N., Oh Y.K., Kim D.W., Kim J.K., Yong C.S., Kim J.O. (2013). Development of docetaxel-loaded solid self-nanoemulsifying drug delivery system (SNEDDS) for enhanced chemotherapeutic effect. Int. J. Pharm..

[37] Balakumar K., Raghavan C.V., Abdu S. (2013). Self-nanoemulsifying drug delivery system (SNEDDS) of rosuvastatin calcium: Design, formulation, bioavailability and pharmacokinetic evaluation. Colloids Surfaces B Biointerfaces.

[38] Zhao Y., Wang C., Chow A.H.L., Ren K., Gong T., Zhang Z., Zhang Y. (2010). Self-nanoemulsifying drug delivery system (SNEDDS) for oral delivery of Zedoary essential oil: Formulation and bioavailability studies. Int. J. Pharm..

[39] Badran M.M., Taha E.I., Tayel M.M., Al-Suwayeh S.A. (2014). Ultra-fine self nanoemulsifying drug delivery system for transdermal delivery of meloxicam: Dependency on the type of surfactants. J. Mol. Liq..

[40] Kyobula M., Adedeji A., Alexander M.R., Saleh E., Wildman R., Ashcroft I., Gellert P.R., Roberts C.J. (2017). 3D inkjet printing of tablets exploiting bespoke complex geometries for controlled and tuneable drug release. J. Control Release.

[41] Ito Y., Kusawake T., Ishida M., Tawa R., Shibata N., Takada K. (2005). Oral solid gentamicin preparation using emulsifier and adsorbent. J. Control Release.

[42] Wang Z., Sun J., Wang Y., Liu X., Liu Y., Fu Q., Meng P., He Z. (2010). Solid self-emulsifying nitrendipine pellets: Preparation and in vitro/in vivo evaluation. Int. J. Pharm..

[43] Ansen T., Holm P., Schultz K. (2004). Process characteristics and compaction of spray-dried emulsions containing a drug dissolved in lipid. Int. J. Pharm..

[44] Buya A.B., Beloqui A., Memvanga P.B., Preat V. (2020). Self-Nano-Emulsifying Drug-Delivery Systems: From the Development to the Current Applications and Challenges in Oral Drug Delivery. Pharmaceutics.

[45] Pouton C.W., Porter C.J.H. (2008). Formulation of lipid-based delivery systems for oral administration: Materials, methods and strategies. Adv. Drug Deliv. Rev..

[46] Jannin V., Musakhanian J., Marchaud D. (2008). Approaches for the development of solid and semi-solid lipid-based formulations. Adv. Drug Deliv. Rev..

[47] Abdelmonem R., Azer M.S., Makky A., Zaghloul A., El-Nabarawi M., Nada A. (2020). Development, Characterization, and in-vivo Pharmacokinetic Study of Lamotrigine Solid Self-Nanoemulsifying Drug Delivery System. Drug Des. Dev. Ther..

[48] Beg S., Katare O.P., Saini S., Garg B., Khurana R.K., Singh B. (2016). Solid self-nanoemulsifying systems of olmesartan medoxomil: Formulation development, micromeritic characterization, in vitro and in vivo evaluation. Powder Technol..

