# Supplementary material for: 3D Printing of Dapagliflozin Containing Self-Nanoemulsifying Tablets: Formulation Design and In Vitro Characterization
Source: Pharmaceutics. 2021 Jun 30;13(7):993. doi: 10.3390/pharmaceutics13070993 (PMC8309195; doi:10.3390/pharmaceutics13070993)
Supplement: Supplementary file 1 [file pharmaceutics-13-00993-s001.zip › pharmaceutics-1260419-supplementary.pdf]

pharmaceutics

Supplementary Materials: 3D Printing of Dapagliflozin Containing Self-nanoemulsifying Tablets: Formulation Design and In Vitro Characterization

Mohammed S. Algahtani, Abdul Aleem Mohammed, Javed Ahmad, M. M. Abdullah and Ehab Saleh

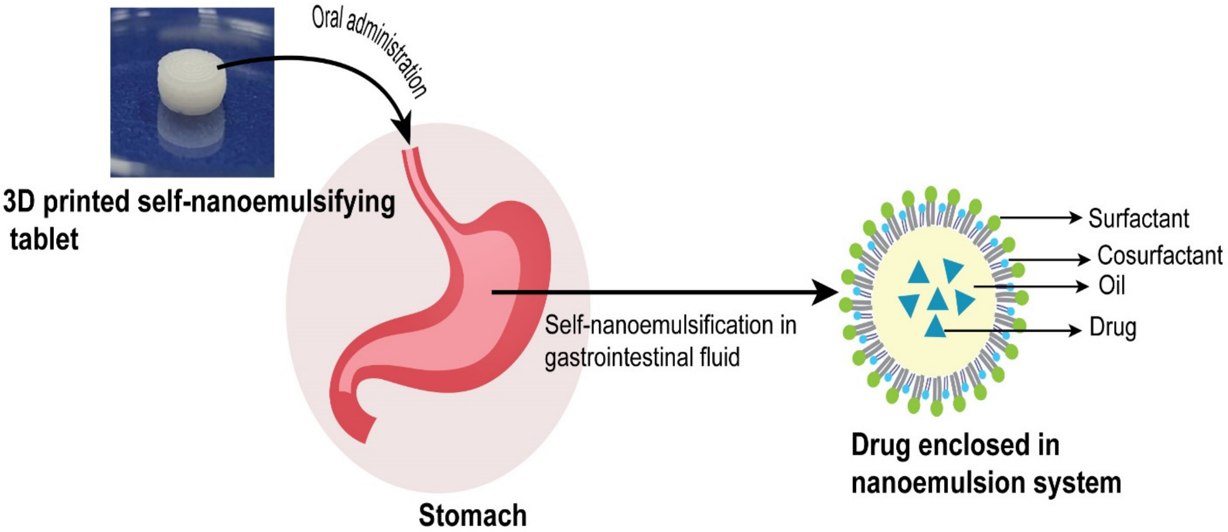

Figure S1. Schematic illustration presenting generation of drug enclosed nanoemulsion system upon self-nanoemulsification of 3D printed self-nanoemulsifying tablet in gastrointestinal fluid.

Table S1. Optimization of extrudability behavior based on printed layer/line dimension (thickness and height).

**Nozzle diameter**	**0.840 mm**	
Printed lines	***Line thickness (mm)***	***Line height (mm)***
Line 1	1.108	0.679
Line 2	0.964	0.669
Line 3	0.898	0.749
Line 4	1.156	0.795
Average ± SD	1.0315 ± 0.12	0.723 ± 0.05
